# The Danish Shoulder Arthroplasty Registry: clinical outcome and short-term survival of 2,137 primary shoulder replacements

**DOI:** 10.3109/17453674.2012.665327

**Published:** 2012-04-24

**Authors:** Jeppe V Rasmussen, John Jakobsen, Stig Brorson, Bo S Olsen

**Affiliations:** ^1^Department of Orthopaedic Surgery, Herlev University Hospital, Herlev; ^2^ARTROS Private Hospital, Aalborg, Denmark

## Abstract

The Danish Shoulder Arthroplasty Registry (DSR) was established in 2004. Data are reported electronically by the surgeons. Patient-reported outcome is collected 10–14 months postoperatively using the Western Ontario osteoarthritis of the shoulder index (WOOS). 2,137 primary shoulder arthroplasties (70% women) were reported to the registry between January 2006 and December 2008. Mean age at surgery was 69 years (SD 12). The most common indications were a displaced proximal humeral fracture (54%) or osteoarthritis (30%). 61% were stemmed hemiarthroplasties, 28% resurfacing hemiarthroplasties, 8% reverse shoulder arthroplasties, and 3% total arthroplasties. Median WOOS was 59% (IQR: 37–82). 5% had been revised by the end of June 2010. The most frequent indications for revision were dislocation or glenoid attrition.

In the 1990s, a group of Danish shoulder surgeons planned a national registry for shoulder replacement. After financing was secured, the Danish Shoulder Arthroplasty Registry (DSR) was established. The purpose is to monitor and improve shoulder arthroplasty surgery. In this paper, we present the DSR.

## Patients and methods

DSR was established in January 2004. At the start, the reporting of information was voluntary but in 2006 the National Board of Health made reporting mandatory. Negligence can result in loss of license to perform shoulder arthroplasty. The registry is located at the Department of Clinical Epidemiology, Aarhus University Hospital, Aarhus; it is financed by the Danish counties and has no dependency on commercial parties.

Data are reported electronically by the surgeon (Appendix). Patient-reported outcomes are collected by mail 10–14 months after surgery using the Western Ontario osteoarthritis of the shoulder index (WOOS). If an arthroplasty is revised, the surgeon reports time, reason, and type of revision. Revision is defined as removal or exchange of part of or the whole arthroplasty, or the addition of a glenoid component to an existing hemiarthroplasty.

WOOS is a 19-question patient-reported outcome for measurement of the shoulder-related quality of life of patients with osteoarthritis of the shoulder (Lo et al. 2001). Each question is answered on a visual analog scale from 0 to 100, with 100 worst. The raw scores are converted to a percentage of the maximum score.

To validate data, shoulder replacements reported to the DSR are compared with data in the Danish National Patient Registry (NPR). Any difference is analyzed and corrected if possible.

### Statistics

Descriptive statistics were used to describe demographic data, patient-reported outcome, and reasons for revision. Kaplan-Meier statistics were used to calculate revision rates with revision for any reason as endpoint. Deaths were checked with the Danish National Register of Persons. We used SPSS software version 17.0.

## Results

2,137 primary shoulder arthroplasties (70% women) were reported to the registry between January 2006 and December 2008. 54 patients had bilateral replacements (with each replacement being considered a separate case). Mean age at surgery was 69 years (SD 12). Compared to the NPR, the DSR had received reports on 88% of the operated patients.

**Figure 1.**
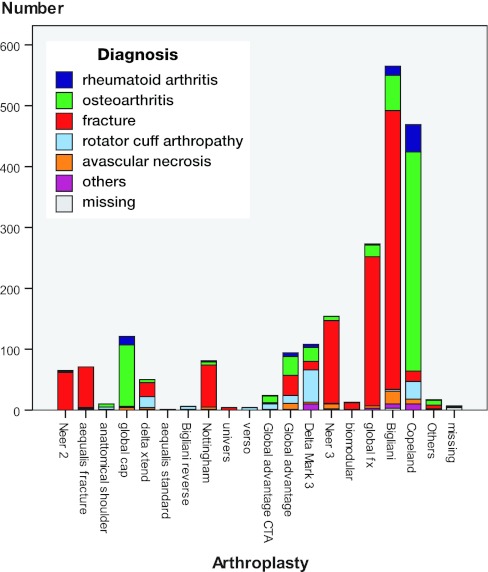
Implants used from January 2006 through December 2008.

**Figure 2.**
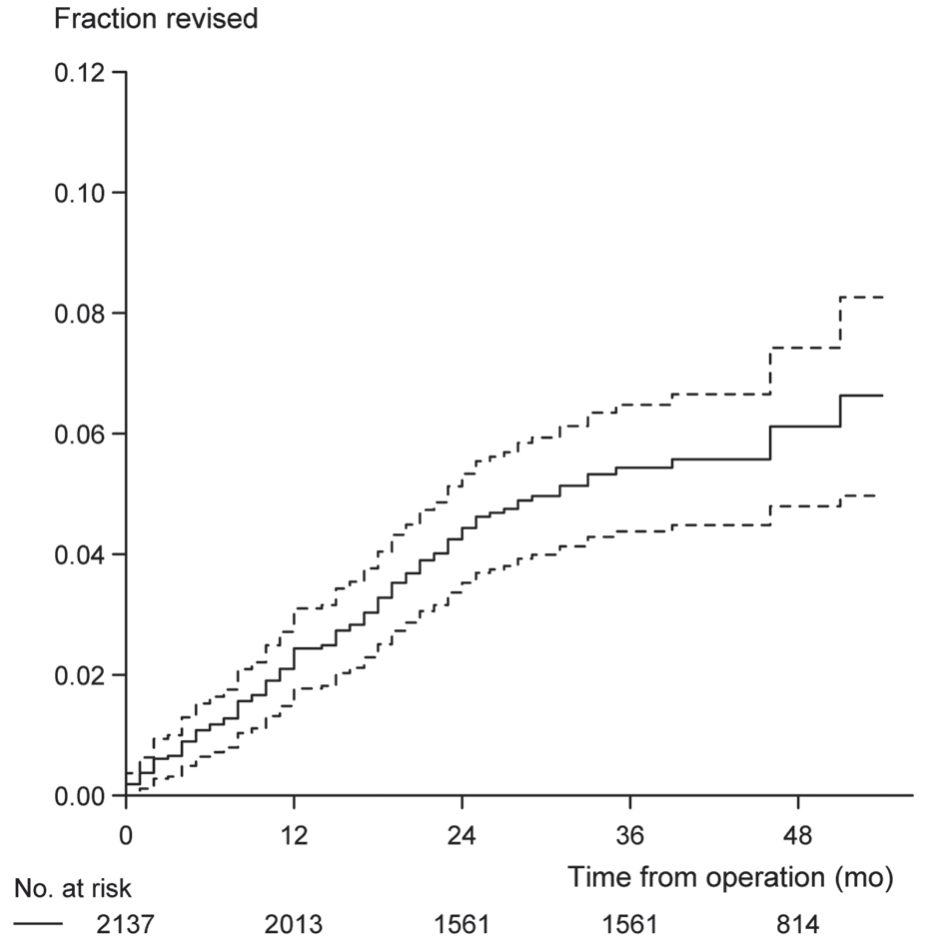
The fraction revised for all arthroplasties reported with 95% CI.

The indications were a displaced proximal humeral fracture (54%), osteoarthritis (30%), rotator cuff arthropathy (7%), rheumatoid arthritis (4%), or avascular necrosis (3%). 61% were stemmed hemiarthroplasties, 28% resurfacing hemiarthroplasties, 8% reverse shoulder arthroplasties, and 3% total shoulder arthroplasties. 19 different implants were used (Figure 1). 76 patients (4%) died within 1 year and could not participate in the follow-up evaluation. 73% of patients returned a questionnaire; however, only 66% of them were complete. Median WOOS for all diagnoses was 59% (IQR 37–82). 5% of the primary arthroplasties between Jan 2006 and Dec 2008 had been revised by the end of June 2010 (Figure 2). The most frequent indications for revision were dislocation (n = 24) or glenoid attrition (n = 18) (Table).

**Table:** Cause of revision

	No.	Percentage of all arthroplasties	Percentage of revisions
Dislocation	24	1.1	22
Loosening	5	0.2	5
Glenoid attrition	18	0.8	17
Infection	10	0.4	9
Technical failure	14	0.6	13
Rotator cuff problem	14	0.6	13
Pain	14	0.6	13
Others	6	0.3	6
Missing	2	0.1	2
Total	107	4.8	100

## Discussion

Compared to the Norwegian and the Swedish registries, rheumatoid arthritis was a rare diagnosis in the Danish Shoulder Arthroplasty Registry (Fevang et al. 2009, Rahme et al. 2001). Since our data were collected more recently, one possible explanation of the different findings might be the reduced need for surgical treatment of patients with rheumatoid arthritis due to improvements in the medical treatment (Fevang et al. 2007).

Total shoulder arthroplasty is seldom used in Denmark compared to the United Kingdom where 31% of all shoulder replacements and 40% of elective shoulder replacements are total shoulder arthroplasties (Ravenscroft and Calvert 2004). The reason is unknown, but the most likely explanation is a tradition of not using a glenoid component due to the risk of aseptic loosening. It is unlikely that the structure of the Danish healthcare system with public financing would have an influence on the use of different shoulder arthroplasty designs.

We found a revision rate of 5% after approximately 5 years. This is comparable to the revision rate reported from the Norwegian registry (Fevang et al. 2009). The most frequent reason for revision in our study were dislocation or glenoid attrition. This is similar to the results from the Norwegian registry (Fevang et al. 2009). In both countries, aseptic loosening including loosening of the glenoid component is less frequently reported probably because of the rare use of total shoulder replacement.

Compared to hip and knee arthroplasties, few shoulder replacements are performed and there are very few data on long-term survival and reasons for revision in the existing literature. The DSR was established with the purpose of monitoring and improving shoulder arthroplasty surgery. It was launched successfully as planned; however, patient compliance remains a challenge. We prepare to send reminders to the patients twice, and if there is still no reply the patient is contacted by telephone. Furthermore, incorrectly filled in questionnaires are returned to the patients to be revised.

The use of revision as a measure of survival of the implant has the advantage of being simple and reliable, but it also has some limitations—especially the fact that a decision to revise depends on several factors such as age, diagnosis, comorbidity, activities of daily living (ADL), and the consent of the patient. The decision can also depend on the ability to convert the failed implant to some other treatment with an expected satisfactory result. Finally and perhaps most importantly, survival of an implant as an outcome measure does not give any information about the majority of arthroplasties that are never revised.

It can be questioned whether WOOS or the Oxford shoulder score (OSS) (Dawson et al. 1996) is the most appropriate patient-reported outcome measure to use for functional outcome in a shoulder arthroplasty registry. When the DSR was established, it was decided to use the same patient-reported outcome as in the Swedish registry, mainly in order to be able to pool data and compare results. Neither WOOS nor OSS has been validated in patients with rotator cuff arthropathy, avascular necrosis, or a proximal humeral fracture treated with shoulder replacement.

As the amount of data increases, the DSR will become a valuable tool for obtaining information on risk factors, patient-reported outcome, and implant survival. It will complement randomized clinical trials and longitudinal studies for the continued improvement of shoulder arthroplasty surgery. To improve the reporting on functional outcome, we are preparing to add a preoperative measurement (baseline setting) for non-traumatic patients and a long-term follow-up measurement. We are also preparing several methodological studies including a validation of the Danish translation of WOOS for both traumatic and non-traumatic patients, and a study describing the demographic properties of non-responders. Finally, we are currently checking the surgeon-reported data by comparing the registry data with the surgical procedures reported in medical journals.

## Appendix

### Data entered in The Danish Shoulder Arthroplasty Register (DSR)

Personal identification number

Name

Date of birth

Gender

Hospital

Date of surgery

Side operated on

Diagnosis: arthritis (rheumatoid, juvenile, psoriatic, others), osteoarthritis (primary, secondary), fracture (time to surgery < 14 days, healed), rotator cuff arthropathy, avascular necrosis, other diagnoses

Prior surgery: none, synovectomy, osteosynthesis, cuff reconstruction, arthroplasty implant, subacromial decompression, extraction of implant, A-C resection, infection, arthroscopic procedure, other

Arthroplasty brand

Arthroplasty design

Stem: modular, monoblock, cemented, uncemented

Caput: conventional, asymmetric, CTA, other

Glenoid: plastic, metal-backed, hybrid, other

Additional surgery: cuff reconstruction, subacromial decompression, A-C resection, bicepstenotomy, biceps tenodese

Type of revision: none, extraction of the humeral component, addition of new humeral component, extraction of the glenoid component, addition of new glenoid component, change of modular head, repositioning of dislocation, infection, refixation of tubercles, osteosynthesis, A-C resection, cuff reconstruction, other

Cause of revision: dislocation, loosening, glenoid attrition, infection, periprosthetic fracture, technical failure, rotator cuff problems, other including pain.
